# Current Limitations and Recent Progress in Nanomedicine for Clinically Available Photodynamic Therapy

**DOI:** 10.3390/biomedicines9010085

**Published:** 2021-01-16

**Authors:** Jooho Park, Yong-Kyu Lee, In-Kyu Park, Seung Rim Hwang

**Affiliations:** 1 Department of Biomedical Chemistry, College of Biomedical and Health Science, Konkuk University, Chungju 27478, Korea; pkjhdn@kku.ac.kr; 2 Department of Chemical and Biological Engineering, Korea National University of Transportation, Chungju 27469, Korea; leeyk@ut.ac.kr; 3 Department of Biomedical Sciences, Chonnam National University Medical School, Hwasun 58128, Korea; pik96@jnu.ac.kr; 4 College of Pharmacy, Chosun University, Gwangju 61452, Korea

**Keywords:** nanomedicine, photodynamic therapy, photosensitizer, nanocarrier, self-assembly, prodrug

## Abstract

Photodynamic therapy (PDT) using oxygen, light, and photosensitizers has been receiving great attention, because it has potential for making up for the weakness of the existing therapies such as surgery, radiation therapy, and chemotherapy. It has been mainly used to treat cancer, and clinical tests for second-generation photosensitizers with improved physicochemical properties, pharmacokinetic profiles, or singlet oxygen quantum yield have been conducted. Progress is also being made in cancer theranostics by using fluorescent signals generated by photosensitizers. In order to obtain the effective cytotoxic effects on the target cells and prevent off-target side effects, photosensitizers need to be localized to the target tissue. The use of nanocarriers combined with photosensitizers can enhance accumulation of photosensitizers in the tumor site, owing to preferential extravasation of nanoparticles into the tumor vasculature by the enhanced permeability and retention effect. Self-assembly of amphiphilic polymers provide good loading efficiency and sustained release of hydrophobic photosensitizers. In addition, prodrug nanomedicines for PDT can be activated by stimuli in the tumor site. In this review, we introduce current limitations and recent progress in nanomedicine for PDT and discuss the expected future direction of research.

## 1. Introduction

The discovery of dyes and their use in combination with light since the end of the nineteenth century led to the idea of modern photodynamic therapy (PDT) for the treatment of cancers, infections, and other diseases [1,2]. PDT has been receiving great attention, because it has potential for making up for the weakness of the existing therapies such as surgery, radiation therapy, and chemotherapy. In order to destroy malignant cells, PDT is done in two stages involving administration of a light-responsive agent known as a photosensitizer and activation of the photosensitizer by light irradiation, usually using a laser [3]. The wavelength of near-infrared (NIR) light is useful for tissue penetration without interference by endogenous chromophores [4]. When photosensitizer molecules are excited by absorption of light, they can react with substrates to form free radicals, or the absorbed photon energy in the photosensitizer molecules is transferred to molecular triplet oxygen after intersystem crossing between the two different electronic states to generate reactive oxygen species (ROS) [5]. The distance diffused by ROS is estimated to be 0.01–0.02 μm. Namely, the lifetime of ROS, mainly of ^1^O_2_, in the cells is estimated to be 0.01–0.04 μs [6].

The singlet oxygen with high reactivity can diffuse across the cell membrane and induce intracellular signaling, resulting in organelle damage and cell death [7].

In order to obtain the effective cytotoxic effects on tumor cells (larger than 10 μm), photosensitizers administered in vivo need to be localized to the target tissue. In the current era of nanotechnology, scientific studies on the use of nanomaterials in PDT are increasing for the treatment of a wide range of diseases, especially cancer. Encapsulation of photosensitizer molecules into the nanocarrier targeting neoplastic endothelial cells can be selectively delivered to the tumor endothelium by the enhanced permeability and retention (EPR) effect [8]. Generation of ROS by irradiation of ultrashort pulse lasers induces cytotoxic effects on vascular endothelial cells and widens intercellular spaces in the blood vessels, improving the efficient accumulation and therapeutic efficacy of anti-angiogenic therapies [9,10]. Since engineered nanomaterials with at least one dimension of around 100 nm have shown great potential as nanocarriers with complementary and supplementary roles in PDT, their use in combination with photosensitizers has increased over the last decade [11].

Pharmacokinetic profiles of NIR photosensitizers together with the prognosis of diseases have also been studied by using NIR imaging [12]. As gold nanorods show the surface plasmon absorption in the NIR region, gold nanorod–photosensitizer complexes were invented as the multifunctional nanoplatform for photodynamic/photothermal therapy as well as fluorescence imaging [13]. Coating multifunctional gold nanorods with the mesoporous silica shell enhances stability of the photosensitizer and the surface plasmon absorption band of gold nanorods [14]. The micellar nanoscale delivery system for a photosensitizer was reported to show theranostic potential for brain tumors [15].

**Figure 1:**
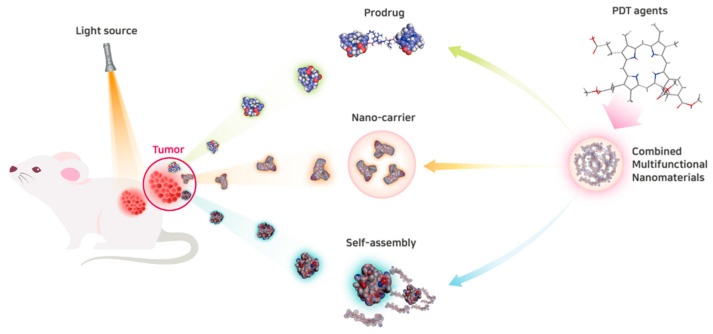
Schematic representation of nanomedicine for photodynamic therapy (PDT). Accumulation of photosensitizers in the tumor site can be enhanced by nanocarriers. In addition, prodrug nanomedicines for PDT can be activated by such stimuli as enzymes in the tumor site. Self-assembly of amphiphilic polymers may provide good loading efficiency and sustained release of hydrophobic photosensitizers.

Human clinical studies on PDT for the treatment of early-stage bladder, skin, lung, esophagus, and stomach cancers showed promising therapeutic responses [16,17,18,19]. However, clinical improvements in cancer patient survival have been observed only at high doses. There are several challenges that must be overcome for the clinical use of PDT agents. Appropriate dosage forms that improve solubility and stability of hydrophobic photosensitizers in biological fluids are required. Nanoparticles formed by self-assembly of amphiphilic copolymers may provide good loading efficiency and sustained release of hydrophobic photosensitizers [20]. In addition, prodrug nanomedicines for PDT can be activated by stimuli such as enzymes in the tumor site (Figure 1).

Therefore, delivery to the specific target site and therapeutic efficacy must be investigated for the clinical use of nanomedicine for PDT. Although several challenges remain to expand the therapeutic application of PDT in the clinic, the advanced nanoplatforms potentially offer the best hope for PDT. In this review, we summarized therapeutic benefits of PDT and current limitations in their development or clinical use. We also discussed recent progress in nanomedicine for PDT as well as the expected future research direction.

## 2. Current Limitations of PDT and Nanomedicine

After photosensitizers combined with nanocarriers are intravenously injected into cancer patients or tumor-bearing mice, they can accumulate in the tumor tissue. They produce ROS only when excited by light of a specific wavelength and exhibit cytotoxic effects on tumor cells. As other normal tissues that have not been exposed to light irradiation are preserved, PDT has the following advantages and disadvantages compared to the existing standard cancer treatment such as surgery or chemotherapy.

### 2.1. Advantages and Disadvantages of PDT in Cancer Treatment

While chemotherapeutic drugs exhibit side effects not only on cancer cells but also on normal cells, photosensitizers combined with nanocarriers selectively accumulate in the tumor site and show cytotoxicity only in the area exposed to light irradiation. PDT can be performed instead of surgery in inoperable patients; light irradiation of the surgical site in patients who underwent tumor removal surgery can decrease the risk of cancer recurrence [21]. Combination of PDT with chemotherapy has the advantage of reducing side effects by lowering the dose of anticancer drugs [22].

However, contrary to systemic chemotherapy, local treatment using an optical fiber in PDT is hard to kill the tumor cells present outside the focal area or alter the therapeutic outcomes in patients with advanced-stage cancer [23]. Under light irradiation for PDT, the penetration efficiency of light into the deep tissue is low because of endogenous biomolecules absorbing the light. It is difficult to efficiently excite the photosensitizers located deeper than 1 cm from the tumor surface. Although there have been recent advances in microendoscopic technology or laparoscopic light delivery systems, PDT still has obvious limitations in treating large, deeply hidden tumors [24].

Among photosensitizer classes such as porphyrins, chlorophylls, and dyes, most photosensitizers approved for clinical use are derivatives of the porphyrin moiety [25]. Porphyrins are composed of tetrapyrrole macrocycles connected to each other via methine bridges [26]. Limitations of the first-generation photosensitizers in clinical application to various solid tumors include aggregation in water, low singlet oxygen quantum yield, high doses needed for therapeutic efficacy, low selectivity to the tumor site, skin photosensitivity, and long elimination half-life [27]. The first-generation photosensitizers based on the porphyrin backbone exhibited undesirable hydrophobicity and low penetration depth, hindering their clinical use. The second-generation photosensitizers such as chlorins and phthalocyanines, which are structurally related to tetrapyrrole macrocycles, have been investigated for cancer treatment [27,28]. Their water solubility, pKa value, and stability were improved by introduction of the hydrophilic substituents to pyrrole rings. However, the increase in renal clearance due to improved water solubility tends to decrease bioavailability of the photosensitizer.

In order to be excited in the deeper tissue, the second-generation photosensitizers have been developed to show higher molar extinction coefficient in the near-infrared region than the first-generation photosensitizers [29]. 5-Aminolevulinic acid (5-ALA), a precursor of protoporphyrin IX, has shown good clinical outcomes in cancer treatment [30,31]. Protoporphyrin IX, an intermediate in the heme biosynthesis, exhibits cytotoxicity when excited by light, and loses phototoxicity by binding to iron ions. Administration of excess exogenous ALA promotes production of protoporphyrin IX more efficiently in cancer cells than in normal cells. One of the disadvantages in clinical application of photosensitizers is a risk of skin photosensitivity. Hydrophobic photosensitizers remain nonspecifically accumulated in the skin and eyes after photodynamic treatment [32]. Contrary to other porphyrin-based photosensitizers, protoporphyrin IX produced by systemic administration of 5-ALA is eliminated after 24–48 h with the lower risk of long-term photosensitivity (Table 1).

The ratio between type I and type II reactions of photosensitizers in cancer depends on the concentration of oxygen and substrates in the tumor microenvironment (TME) [33]. When exposed to light of a specific wavelength, photosensitizer molecules are excited to the singlet energy state. While some of the excited photosensitizer molecules return to the ground state by emitting energy in the form of fluorescence, most of the excited molecules transfer to the triplet energy state via intersystem crossing. In type I reaction, the transfer of electrons or hydrogen atoms between photosensitizer molecules excited by light and the surrounding substrates generates radical species, resulting in cytotoxicity through oxidation reaction with intracellular components. In type II reaction, photosensitizer molecules in the triplet state efficiently transfer energy to the surrounding oxygen and generate singlet oxygen [34]. Singlet oxygen induces cytotoxicity via chemical reaction with intracellular components. It is important to consider that the production of cytotoxic ROS in the TME induces oxygen depletion in the tumor tissue, resulting in apoptosis or necrosis in the targeted tissue. After intravenous injection, porphyrin derivatives accumulate in the tumor vasculature, and occlusion by damage of vascular epithelial cells occurs under light irradiation [35]. It induces hypoxia and tumor necrosis. As therapeutic outcomes of PDT are dependent on the preexisting concentration of oxygen at the tumor site, strategies are needed for use of PDT in the treatment of hypoxic tumors [36].

Effective cytotoxic effects can be obtained by localization of photosensitizers in the intracellular organelles such as mitochondria, nucleus, and lysosomes because of reactivity and short half-life of the ROS generated by light irradiation [37]. In order to allow photosensitizers to accumulate in the target site at sufficient concentrations, modification of photosensitizers has been attempted by using cell-penetrating peptides or conjugation with targeting ligands [38]. Besides, therapeutic efficacy of PDT depends on the wavelength of light, laser power per unit area, and the dosage of photosensitizers [39]. Over the past few decades, extensive attention has been paid to the design and development of various PDT modalities.

### 2.2. Current Limitations in Clinical Application of Nanomedicine

Rapid progress in the field of nanotechnology facilitates control of physicochemical properties such as the diameter, shape, and surface functional group of nanomaterials. It has enabled development of nanomedicines that respond to stimuli including light irradiation and the expansion of PDT strategies in conjunction with nanomedicine [40]. The use of nanoparticles has the advantage in the prolonged circulation and accumulation in the tumor site owing to the EPR effect, which depends on the leaky vasculature of solid tumors [41]. The tumor vasculature also contributes to the immunosuppressive TME, and cancer cells communicate with TME components including immune cells through the excess of vasculature mediators [42]. Advanced nanocarriers grafted with a targeting moiety have been designed to specifically interact with target cells or actively target cargo drugs, which would be helpful in minimizing off-target toxicity to healthy cells and increasing therapeutic efficacy [43,44]. Encapsulation of drugs within nanoparticles has advantages in protection or controlled release of cargo drugs, and pharmacokinetics of nanomedicines are dependent on the physicochemical characteristics of nanocarriers [45]. To date, already approved drugs are preferable to newly investigated drug candidates in the nanoformulation of oncology drugs, and clinical trials and FDA approvals for nanomedicines are still limited. Nanomedicines such as PEGylated liposomal doxorubicin (Doxil^®^), albumin-bound paclitaxel (Abraxane^®^), and PEGylated proteins are used in the clinical setting [46]. In phase I clinical studies, they showed a greater ratio of the maximum tolerated dose to the starting dose than small-molecule drugs [47]. It suggests that the appropriate preclinical model is required for evaluating toxicity of nanomedicines.

Preferential accumulation of cancer therapeutics in the tumor might be achieved by using stimuli-responsive nanoparticles with targeting ligands [48]. Distribution of nanoparticles in the intratumoral environment was reportedly analyzed by using fluorescent particles [49]. However, whole body imaging of nanoparticles using NIR fluorophores varies according to the dye content and concentration of nanoparticles and often leads us to draw improper conclusions [50]. In addition, most anti-cancer nanomedicines show their heterogeneous accumulation in the tumor and limited clinical outcomes because of the failure to overcome the realistic physiological transport barriers and inter-patient variability [51]. Despite advances in technologies for the targeting of therapeutic nanoparticles to the tumor tissue, less than 1% of intravenously injected nanoparticles normally reach the tumor [50]. Nanomedicines targeting specific molecules that are generally overexpressed on the surface of cancer cells or in the TME have not yet reached the market owing to the complexity and heterogeneity of tumors in the body. Hence, it is increasingly evident that modern nanomedicines for PDT should overcome the limitations of traditional nanomedicines, such as low delivery efficacy and poor clinical outcomes, using novel strategies.

## 3. Advances in Nanomedicine for PDT to Overcome Current Clinical Limitations

### 3.1. Advances in Nanocarriers for PDT

To date, numerous nanocarriers such as polymers, micelles, liposomes, dendrimers, and inorganic nanoparticles have been studied for increasing therapeutic efficacy of photosensitizers. It is important to efficiently deliver photosensitizers and the generated singlet oxygen to the target site in the optimum therapeutic range. Pharmacokinetic or pharmacodynamic profiles of nanocarriers should also be checked for clinical use. Multifunctional nanoparticles are also currently being investigated for theranostic purpose or photodynamic/chemo dual therapy. Recent advances in preclinical developments using nanomedicine for PDT are categorized in Table 2.

Natural polysaccharides such as hyaluronic acid (HA), heparin, chitin, chitosan, and fucoidan have been reported as potential photosensitizer carriers owing to their biocompatibility and biodegradability [69,70,71]. HA shell can interact with the core, including chlorin e6 (Ce6) and positively charged CRISPR–Cas9 targeting the phosphatase gene, which constructs a nanocarrier system [52]. The negatively charged HA in the nanoparticles could not only regulate the surface charge of the nanoparticles reducing nonspecific interactions in the physiological environment, but also target the TME via CD44 receptors [72,73]. This multifunctional nanosystem demonstrated high transfection efficiency in B16F10 cells and PDT efficacy under laser irradiation. It can also sensitize the targeted tumors to immunotherapy by promoting the proliferation of cytotoxic CD8^+^ T cells.

Newly developed biocompatible nanocarriers not only encapsulate PDT agents, but also provide additional targeting effects for enhancing anticancer activities. Fucoidan exhibits binding affinity to P-selectin and the targeting effect on P-selectin-positive cancer cells [74,75]. Chung et al. developed a multifunctional nanocomplex carrying photosensitizer verteporfin, which was composed of negatively charged fucoidan, positively charged polyamidoamine (PAMAM) dendrimer, and MnO_2_ catalyzing the decomposition of hydrogen peroxide to form oxygen. The nanocarrier can enhance accumulation of verteporfin in the tumor site through both P-selectin targeting and the EPR effect. The dendrimer–fucoidan nanocomplex could specifically target P-selectin-overexpressed breast cancer and tumor-associated vasculature [53]. It also overcame tumor hypoxia using MnO_2_ and improved the therapeutic efficacy of PDT for antimetastatic effects. Recent multifunctional nanoplatforms exhibit such characteristics as cancer targeting, improved pharmacokinetics/pharmacodynamics, and high photodynamic efficacy.

In order to overcome drawbacks of chemotherapy, nanocarriers for the combination of chemotherapy and PDT have also been developed. Ce6 was loaded into peroxidase mimic metal–organic nanoparticles and coated with HA [54]. The nanocarrier can react with hydrogen peroxide in the tumor site and form oxygen to prevent hypoxia. It was designed for exhibiting cascade reactions and it could show synergetic chemo–photodynamic therapeutic efficacy.

Attachment of polyethylene glycol to the nanocarrier can provide hydrophilicity, decrease the clearance by the reticuloendothelial system, and extend circulation time. Polyethylene glycol (PEG) conjugated with hydrophobic stearamine carrying doxorubicin and pheophorbide A (PhA) accumulated in the tumor site, and the release of drugs from the nanocarrier with a ROS-sensitive linker was triggered by ROS within cancer cells [55]. The outer PEG layer could be helpful for long circulation of the nanocarrier in blood, and a stable thioketal bond could prevent premature drug leakage. On the other hand, there is growing concern about the immune response induced by PEGylation. Injection of PEGylated multifunctional nanoplatforms or bioconjugates may induce immunogenic reaction in the body [76]. Alternatively, zwitterionic polymers that have a hydrophilic nature and excellent biocompatibility can be used in the nanocarriers [77,78,79,80].

### 3.2. Self-Assembly of Nanomedicine for PDT

As self-assembled polymeric micelles or liposomes have amphiphilic structures in an aqueous solution, hydrophobic photosensitizers can be loaded into the hydrophobic core space. Biocompatible phospholipids or polymers approved by the FDA used in self-assembly of organic nanomedicine are dissociated with cargo photosensitizers after being endocytosed into the target cells and offer no severe toxicity by themselves. Yoon et al. reported that Ce6, a hydrophobic photosensitizer, was loaded into amphiphilic HA nanoparticles grafted with hydrophobic 5β-cholanic acid groups, PEG, and Black Hole Quencher 3 [81]. It showed drug loading efficiency higher than 80%, and the self-assembled hyaluronic acid nanoparticles were degraded by hyaluronidases in tumor cells after cellular uptake of particles.

Photosensitizers conjugated to polymers could also be self-assembled. Ce6 conjugated to mPEG-grafted HA formed self-assembled nanoparticles, in which camptothecin was encapsulated for photodynamic/chemo dual therapy [56]. When anticancer camptothecin is encapsulated in the photosensitizer-conjugated HA nanoparticle, NIR fluorescence and singlet oxygen generation from the nanoparticles can be activated by intracellular hyaluronidase and exert theranostic potential for triple-negative breast cancer. Luo et al. reported the “one-pot” fabrication approach using polyvinyl alcohol (PVA)–porphyrin compounds which were synthesized by esterification between polymers and photosensitizers [57]. Hydrophobic doxorubicin loaded into amphiphilic PVA–porphyrin nanoparticles showed 53 times longer half-lives than free doxorubicin in rats. Metal ions for positron emission tomography could also be chelated to photodynamic porphyrin structures in doxorubicin-loaded nanoparticles. Recently developed drug delivery systems based on functionalized self-assembled nanomaterials have attracted considerable attention.

Inorganic materials which are size-adjustable and large in surface area also show potential as self-assembled nanoparticles for PDT. The structure of the self-assembled porphyrin core–shell nanocomposite surrounded by a silica shell was constructed by the surfactant micelle and the silicate sol–gel process [58]. Kinetics of porphyrin self-assembly and rigidity of the core–shell silica nanoparticles were dependent on pH. It enables efficient energy transfer and high yield of singlet oxygen generation.

**Figure 2:**
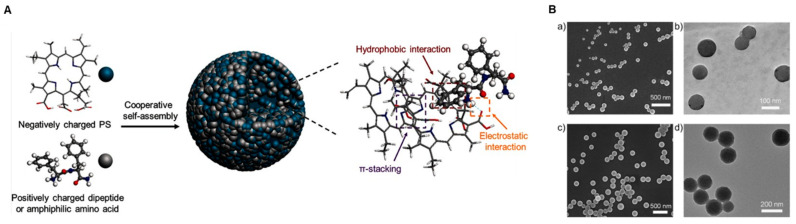
(**A**) A scheme for fabrication of photosensitive nanoparticles by amphiphilic dipeptide- or amino acid-tuned self-assembly. (**B**) (**a**) Scanning electron microscopy (SEM) image and (**b**) transmission electron microscopy (TEM) image of assembled Fmoc-_L_-Lys/Ce6 nanoparticles using Fmoc-_L_-Lys (2.0 mg mL^−1^) and chlorin e6 (Ce6) (0.5 mg mL^−1^) as building blocks. (**c**) SEM image and (**d**) TEM image of assembled cationic diphenylalanine (CDP)/Ce6 nanoparticles using CDP (2.0 mg mL^−1^) and Ce6 (0.5 mg mL^−1^) as building blocks (reproduced with permission from [59]).

Meanwhile, peptides or proteins are also promising candidates for modulating self-assembly of phototherapeutic nanoparticles; they exhibit structural or functional diversity and high biocompatibility or biodegradability [82,83]. Self-assembly of photosensitizers could be tuned by a dipeptide or amphiphilic amino acid, resulting in the enhanced PDT efficacy in mice. Hydrophobic Ce6 was combined with amphiphilic fluorenylmethoxycarbonyl-l-lysine [59]. It undergoes multiple intermolecular interactions, including electrostatic force and π–π stacking interactions; therefore, it can mimic the self-assembly behavior of proteins in the solution (Figure 2). In the porphyrin rings of photosensitizers, there is the interaction caused by π-electrons or van der Waals forces. Amphipathic properties of natural proteins make self-assembled PDT agents promising candidates for biomedical applications by improving the poor pharmacokinetics and pharmacodynamics of free photosensitizers.

In addition, self-assembled nanomaterials are occasionally formed in different sizes, which facilitating deep penetration of photosensitizers or chemo drugs into the tumor. A dye–chemo drug conjugate was reported to enhance the photodynamic efficacy in its molecular self-assembly [60]. The size of the compound of indocyanine, camptothecin, and cyclic arginine–glycine–aspartic (RGD) tripeptide was decreased at the nanoscale by aggregation of dye and intersystem crossing under light irradiation. It allowed deep penetration of the nanodrug into the tumor and distinct singlet oxygen quantum yield, resulting in enhanced theranostic performance.

**Figure 3:**
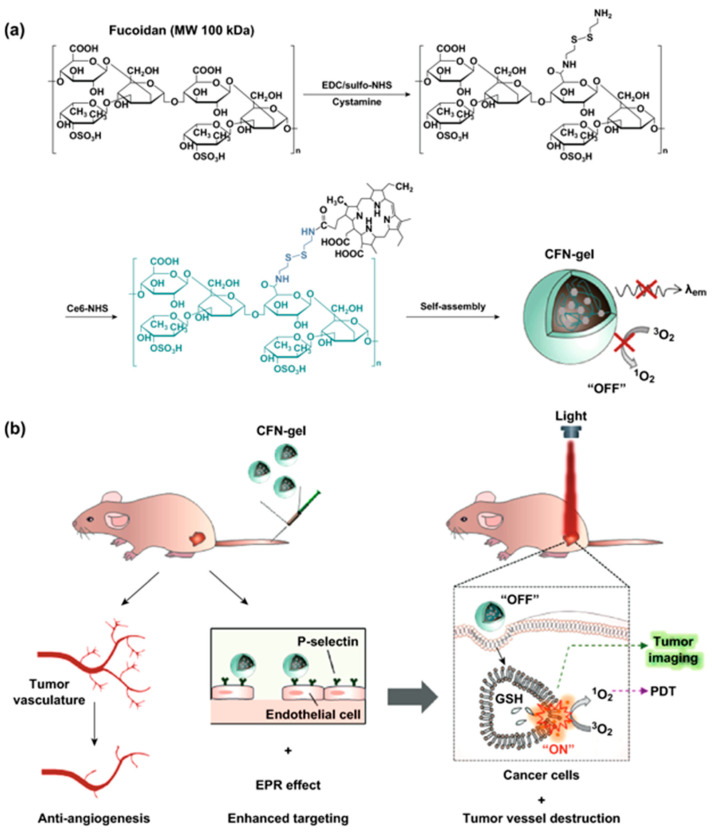
(**a**) Synthesis of the C36–fucoidan theranostic nanogel (CFN-gel). (**b**) schematic illustration of the CFN-gel and its mode of action. EDC: 1-ethyl-3-(3-dimethylamino) propyl carbodiimide; NHS: N-hydroxysuccinimide; GSH: glutathione (reproduced with permission from Cho, M.H.; Li, Y.; Lo, P.C.; Lee, H.R.; Choi, Y. Fucoidan-Based Theranostic Nanogel for Enhancing Imaging and Photodynamic Therapy of Cancer. *Nano Micro. Lett.* **2020,** *12*).

In order to boost the ROS generation efficiency of photosensitizers with aggregation-induced emission (AIE), biocompatible polymers with stimuli-responsive bonds could be self-assembled into nanomicelles in water [61]. Recently, the self-assembled nanogel using Ce6–fucoidan conjugates was reported to induce cancer cell death at the targeted site and fluorescent imaging of tumors in response to the intracellular redox potential [62]. Fucoidan in this nanogel not only carries Ce6, but also exhibits the targeting effect on P-selectin overexpressed on the surface of neovascular endothelial cells (Figure 3).

### 3.3. Stimuli-Responsive Prodrug Nanomedicine for PDT

In recent years, efforts have been made to develop photosensitizers in which the singlet oxygen generation efficiency is suppressed in normal cells and increased only in the target tissue. Use of the nanomedicine that activates singlet oxygen generation only in the tumor site is expected to kill cancer cells while suppressing skin photosensitivity reactions. Stimuli-responsive prodrug nanomedicines have been developed for PDT.

**Figure 4:**
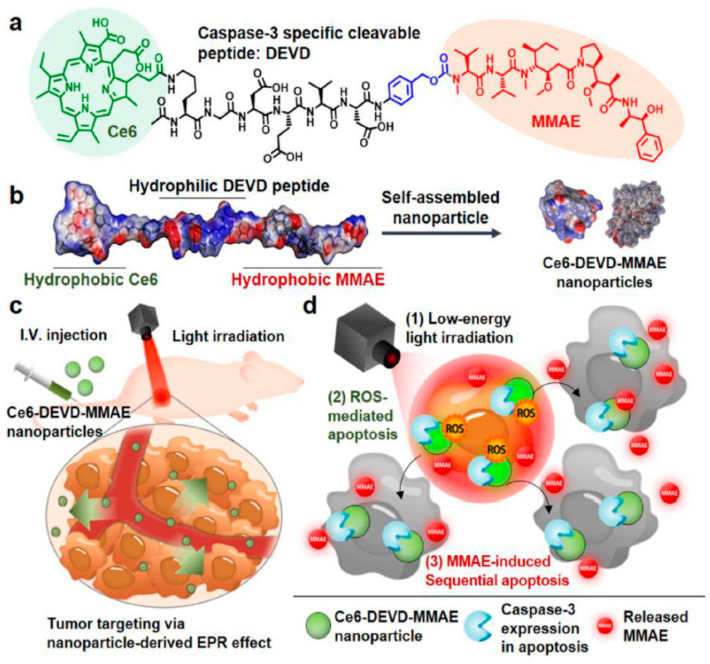
Schematic representation of visible light-induced apoptosis activatable nanoparticles of chlorin e6 (Ce6)–Asp–Glu–Val–Asp (DEVD) –monomethyl auristatin E (MMAE) for targeted cancer therapy. (**a**) The molecular structure of Ce6–DEVD–MMAE consisting of Ce6 (green), DEVD (black), p-amino-benzyl carbamate linker (blue), and MMAE moieties (red). (**b**) The Ce6–DEVD–MMAE can form stable nanoparticles via self-assembly of an amphiphilic prodrug-based structure. (**c**) The self-assembly of Ce6–DEVD–MMAE nanoparticles may enhance drug delivery to targeted tumors via enhanced permeation and retention effect. (**d**) The cytotoxicity of nanoparticles at the targeted tumors can be continuously induced with caspase 3 following exposure to visible light irradiation and it can be further amplified by activating MMAE from Ce6–DEVD–MMAE nanoparticles without visible light irradiation, resulting in sequential, repetitive, and amplified cell death of targeted tumor tissues (reproduced with permission from Um, W.; Park, J.; Ko, H.; Lim, S.; Yoon, H.Y.; Shim, M.K.; Lee, S.; Ko, Y.J.; Kim, M.J.; Park, J.H.; Lim, D.K.; Byun, Y.; Kwon, I.C.; Kim, K. Visible light-induced apoptosis activatable nanoparticles of photosensitizer-DEVD-anticancer drug conjugate for targeted cancer therapy. *Biomaterials* **2019**, *224*, 119494).

A prodrug conjugate between photosensitizer molecules and anticancer drug molecules with the caspase 3-cleavable peptide linker, Asp–Glu–Val–Asp (DEVD), was designed for targeted cancer therapy [84]. Under minimal irradiation intensity, self-assembled Ce6–DEVD–monomethyl auristatin E (MMAE) nanoparticles accumulated in the target tumor tissue and induced sequential apoptosis, because the prodrug could be activated into cytotoxic MMAE by enzyme cleavage of DEVD [63] (Figure 4). This amphiphilic conjugate formed stable self-assembled nanoparticles in the saline without other additional nanocarriers and showed strong efficacy and low systemic toxicity. It has potential for overcoming the current challenges of PDT associated with limited tissue penetration and oxygen depletion in solid tumors.

PEG was also used in prodrug nanomedicine formulations. Kim et al. reported ROS-sensitive prodrug nanoparticles for the combination of chemotherapy and PDT [64]. Self-assembly of doxorubicin conjugated to PEG via the thioketal linker could encapsulate hydrophobic PhA, and the nanoparticles released drugs by ROS in the tumor site, thereby minimizing off-target toxicity. Addition of the active targeting moiety to prodrug systems can enhance drug delivery to the target site for glioma treatment. The chemotherapeutic camptothecin–PEG conjugate via a disulfide bond was further modified with an internalizing RGD peptide and loaded with photosensitizer IR780 [65]. This prodrug efficiently penetrated the blood–brain barrier and was targeted to glioma cells, resulting in effective combination of chemotherapy with PDT.

**Figure 5:**
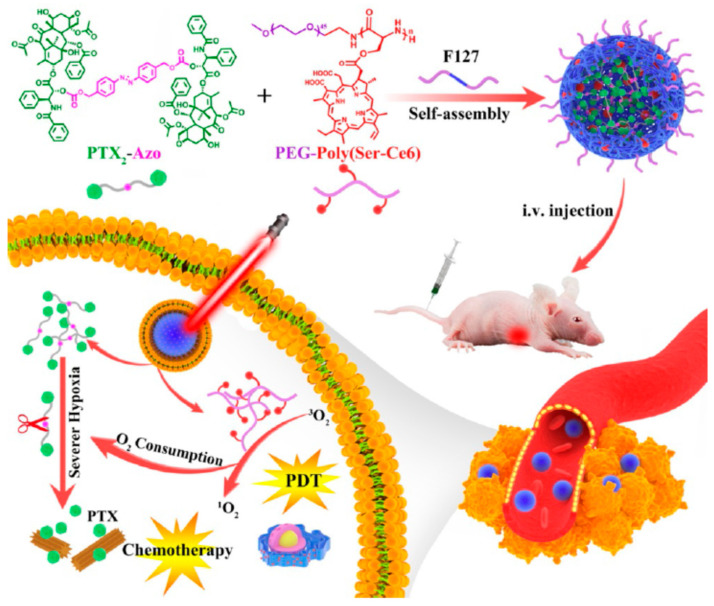
Schematic illustration of the components of the activatable prodrug nanoparticle and its light-boosted hypoxia-activated self-immolative drug release for synergistic tumor inhibition. A light-boosted hypoxia-activated self-immolative paclitaxel (PTX) prodrug nanosystem was designed for synergistic photodynamic therapy and chemotherapy. After intravenous administration, the nanoparticle could gather at the tumor site. Upon irradiation, severe hypoxia occurred and amplified the specific release of paclitaxel from the prodrug bridged with azobenzene. The nanoparticle showed superior antitumor efficacy with little toxicity to other organs (reproduced with permission from Zhou, S.; Hu, X.; Xia, R.; Liu, S.; Pei, Q.; Chen, G.; Xie, Z; Jing, X. A paclitaxel prodrug activatable by irradiation in a hypoxic microenvironment. *Angew. Chem. Int. Ed. Engl.* **2020,** *59*, 23198–23205).

Chemo drugs can be dimerized by the stimuli-responsive chemical linkage for controlled drug release from prodrug nanoparticles. Paclitaxel encapsulated in the PEGylated peptide copolymer combined with a photosensitizer was reported to be activatable by light irradiation as light-boosted nanomedicines [66]. The prodrug nanoparticles generated ROS under light irradiation and promoted drug release in hypoxic TME. The paclitaxel released from the nanoparticles could bind to microtubules along with the ROS produced by Ce6 after irradiation and inhibit cell division, showing synergistic inhibition of tumor growth (Figure 5). The anticancer cabazitaxel dimer with a ROS-activatable thioketal linkage was also developed by co-assembly with Ce6 and formed colloidal stable nanoparticles [67]. Under NIR laser irradiation, the prodrug was activated, and the ROS generated by Ce6 showed synergistic antitumor effects with chemotherapy. The nanoparticles prepared with cabazitaxel prodrug completely eradicated tumors in three of the six melanoma patient-derived xenograft (PDX) mouse models. Polyprodrug approaches were also reported. PhA, a PDT agent, was encapsulated in the core of self-assembled polygalactose-co-poly(cinnamaldehyde). After the polyprodrug nanoparticles were targeted to galactose receptors expressed on tumor cells and endocytosed, cinnamaldehyde and PhA synergistically stimulated ROS generation [68].

Overall, the combination of a prodrug and a PDT agent is promising to achieve maximized therapeutic efficacy with lower toxicity for the successful clinical application of PDT. Prodrug nanomedicines can compensate for the limited efficacy of the ROS induced by light irradiation and PDT agents with low toxicity.

## 4. Conclusions and Future Perspectives

Owing to minimally invasive characteristics, PDT is expected to be more widely applied by replacing surgery or chemotherapy for primary cancer lesions [85]. It may be applied instead of surgery for laryngeal cancer or cervical cancer to preserve vocal or reproductive function [86]. It is worth applying PDT to pancreatic cancer patients who do not respond to chemotherapy and show low survival rate due to difficulty of early detection [87]. Incorporating a soft robot system for an operable laparoscope with PDT would be helpful for PDT in peritoneal organs [88]. With the development of technologies for endoscopy and laparoscopy, indications of PDT have been expanded to various carcinomas. Since PDT has shown various advantages, it is expected to be widely applied not only to cancer treatment, but also to skin diseases, ophthalmic diseases, and neovascular diseases.

The combination of chemotherapy with PDT has thrived as an efficient clinically available strategy to enhance the anticancer therapeutic efficacy and minimize the systemic toxicity of chemotherapy by reducing dose. Besides combination with chemotherapy, combination of PDT with immunotherapy is also being investigated. PDT-mediated destruction of local tumors causes infiltration of leukocytes and stimulates the immune system [89]. As PDT has been shown to induce immunogenic cell death and modulate the TME, a combination of the nanoparticle loaded with a photosensitizer and tumor-specific cancer vaccine would potentiate tumor suppression by immune checkpoint inhibitors [89,90]. EPR effects on photosensitizer-loaded nanoparticles would be improved by molecular targeting or remodeling of the TME combined with light irradiation [91]. PDT shows limited efficacy against hypoxic tumors, because it is dependent on oxygen. Microvessels are also damaged by tumor cell ablation in PDT [36]. In order to overcome hypoxic TME, hypoxia-activatable anticancer prodrug was reportedly co-delivered with a water-soluble photosensitizer using phthalocyanine/albumin supramolecular complexes [92]. During PDT, epidermal growth factor receptor (EGFR) tends to be activated. Thus, PDT is usually more proper in solid tumors than in metastatic tumors. Photosensitizer–EGFR inhibitor conjugate is expected to block treatment escape pathways for PDT [93].

Lack of water solubility or target specificity of photosensitizers can be supplemented by smart nano-enabled delivery systems. Controlled release nanocarriers and targeted photosensitizers are also being continuously developed. Nanomedicines that integrate characteristics of nanocarriers, self-assembled nanoparticles, and stimuli-responsive prodrugs are also emerging. Hydrophobic photosensitizers, for example, conjugated to hydrophilic carbohydrate derivatives through a ROS-responsive linker can be self-assembled into nanoparticles. When nanocarriers reach the tumor site, photosensitizers are be released from the nanoparticles by the ROS-responsive bond cleavage [94].

As the molecular imaging technology advances and is applied to the clinics, the diagnosis and treatment of cancer at the early stage become more important. There has been a lot of research on theranostic nanomedicines for PDT [95]. We may use AIE for image-guided photodynamic killing of cancer cells [96]. Aggregation of fluorophore molecules causes quenching or decrease in fluorescence. However, when luminogens are aggregated and become emissive, fluorescence occurs emergently [97]. Therapeutic effects of luminogen–porphyrin conjugates need to be evaluated in terms of whether the luminogen can pass the excitation energy onto porphyrin rings [98]. We may even try chemiluminescence-guided cancer therapy in order to overcome the limitation of light penetration depth [99]. In addition, PDT using two-photon excitation that refers to the simultaneous absorption of two photons to reach an excited state has shown promising properties including deep-tissue penetration, spatial selectivity with reduced side effects, and remarkable therapeutic efficiency [100,101]. It can be used in combination with photosensitizers with AIE characteristics for overcoming the limitations of conventional single-photon PDT. For the clinical use of nanomedicines in PDT, platform technologies for pharmacokinetic/pharmacodynamic evaluation must be set up. Advances in the development of optical equipment with high-powered lasers and free wavelength selection will enable a large number of patients to benefit from PDT. Application of ultrashort pulse lasers and optimization of light irradiation protocols are also needed for obtaining ideal phototherapeutic efficacy.

## Figures and Tables

**Table 1:** Recent outcomes of clinical trials with photosensitizers.

Photosensitizer	Other Name (s)	Indications	Clinical Trial
Photofrin	Porfimer sodium	Esophageal cancer, endobronchial cancer, high-grade dysplasia in Barrett’s esophagus	FDA-approved PDT drug
Photofrin	Porfimer sodium	Clinical trials (phases I–II) for various cancers	NCT00054002 NCT00003788 NCT00118222 NCT00322699 (Completed ^1^)
Temoporfin	Foscan	Head and neck cancer	-
Temoporfin	Foscan	Clinical trials (phase II)	NCT00003856 (Unknown ^2^)
5-Aminolevulinic acid	5-ALA	Malignant gliomas	For the guiding agent, not therapeutics
Talaporfin	Mono-l-aspartyl chlorin e6, Laserphyrin	Lung cancer	Approved in Japan
Verteporfin	Visudyne	Age-related macular degeneration, subfoveal choroidal neovascularization	Clinical trials for macular degeneration (NCT02081339)

^1^ The studies were completed between 2010 and 2018. ^2^ The study has passed its completion date, and its recruitment status has not been confirmed within the past 2 years.

**Table 2:** Recent advances in preclinical developments using nanomedicine for PDT.

Class	Photosensitizer	Nanomaterial	Highlight	Year	Reference
Nanocarrier	Chlorin e6 (Ce6)	Hyaluronic acid (HA)–based nanomaterials	CRISPR–Cas9 system targeting the Ptpn2 gene	2020	[52]
Nanocarrier	Verteporfin	Dendrimer–fucoidan nanocomplex	Sensitive to glutathione-targeted P-selectin	2020	[53]
Nanocarrier	Ce6	HA shielding could endow nanoplatform	Peroxidase mimic metal–organic framework	2020	[54]
Nanocarrier	Pheophorbide A (PhA)	Polyethylene glycol (PEG)–stearamine	Long-circulating, photodynamic/chemo dual therapy	2020	[55]
Self-assembly	Ce6	mPEG-grafted HA	Photodynamic/chemo dual therapy	2015	[56]
Self-assembly	Porphyrin	Polyvinyl alcohol	One-pot fabrication, theranostics	2017	[57]
Self-assembly	Porphyrin	Silica	pH-Dependent assembly of porphyrin–silica nanocomposites	2017	[58]
Self-assembly	Ce6	Peptide conjugate	Dipeptide and Ce6 conjugate	2016	[59]
Self-assembly	Indocyanine	Pentamethine indocyanine	Indocyanine, camptothecin, RGD peptide	2018	[60]
Self-assembly	MeTTMN	mPEG-SS-OH and cinnamic acid conjugate	Photosensitizers with aggregation-induced emission characteristics	2020	[61]
Self-assembly	Ce6	Fucoidan	Theranostic nanogel	2020	[62]
Prodrug	Ce6	DEVD–monomethyl auristatin E conjugate	Caspase 3-cleavable photosensitizer–drug conjugate	2019	[63]
Prodrug	PhA	PEG–doxorubicin conjugate	Reactive oxygen species (ROS)-sensitive nanoparticles for photodynamic/chemo dual therapy	2020	[64]
Prodrug	IR780	PEG with further modification of internalizing RGD peptide	Camptothecin prodrug for blood–brain barrier penetration	2020	[65]
Prodrug	Ce6	Dimeric paclitaxel encapsulated by a PEGylated peptide copolymer	Prodrug activatable via irradiation in a hypoxic microenvironment	2020	[66]
Prodrug	Ce6	Dimeric cabazitaxel	Cabazitaxel prodrug for photodynamic/chemo dual therapy against melanoma	2020	[67]
Prodrug	PhA	Polygalactose-co-poly-cinnamaldehyde	ROS generation by polyprodrug for combinational therapy	2020	[68]

Ce6: chlorin e6; HA: hyaluronic acid; CRISPR: clustered regularly interspaced short palindromic repeats; PhA: pheophorbide A; PEG: polyethylene glycol; RGD: arginine-glycine-aspartic; DEVD: Asp-Glu-Val-Asp; ROS: reactive oxygen species.

## Data Availability

Data sharing not applicable.
